# The importance of vitamin B_12_ for individuals choosing plant-based diets

**DOI:** 10.1007/s00394-022-03025-4

**Published:** 2022-12-05

**Authors:** Ali Niklewicz, A. David Smith, Alison Smith, Andre Holzer, Andrew Klein, Andrew McCaddon, Anne M. Molloy, Bruce H. R. Wolffenbuttel, Ebba Nexo, Helene McNulty, Helga Refsum, Jean-Louis Gueant, Marie-Joe Dib, Mary Ward, Michelle Murphy, Ralph Green, Kourosh R. Ahmadi, Luciana Hannibal, Martin J. Warren, P. Julian Owen, on behalf of CluB-12

**Affiliations:** 1 Department of Nutritional Sciences, School of Biosciences and Medicine, University of Surrey, Guildford, GU2 7XH UK; 2 OPTIMA, Department of Pharmacology, University of Oxford, Oxford, UK; 3 Department of Plant Sciences, University of Cambridge, Downing Street, Cambridge, CB2 3EA UK; 4 Department of Anaesthesia and Intensive Care, Royal Papworth Hospital, Cambridge, UK; 5 Faculty of Social and Life Sciences, Wrexham Glyndwr University, Wrexham, UK; 6 School of Medicine, Trinity College Dublin, Dublin, Ireland; 7 Department of Endocrinology, University of Groningen, University Medical Center Groningen, HPC AA31, P.O. Box 30001, 9700 RB Groningen, The Netherlands; 8 Department of Clinical Biochemistry, Aarhus University Hospital, Aarhus, Denmark; 9 Nutrition Innovation Centre for Food and Health (NICHE), School of Biomedical Sciences, Ulster University, Coleraine, Northern Ireland UK; 10 Department of Nutrition, Institute of Basic Medical Sciences, University of Oslo, Oslo, Norway; 11 Department of Hepato-Gastroenterology, University Regional Hospital of Nancy, and Inserm UMRS 1256 N-GERE (Nutrition-Genetics-Environmental Risks)-University of Lorraine, 54000 Nancy, France; 12 Department of Epidemiology and Biostatistics, School of Public Health, Imperial College, London, UK; 13 Facultat de Medicina I Ciències de La Salut, Unitat de Medicina Preventiva I Salut Pública, Universitat Rovira I Virgili, Reus, IISPV, CIBEROBN, Tarragona, Spain; 14 School of Medicine, University of California, Davis, CA USA; 15 Laboratory of Clinical Biochemistry and Metabolism, Department of General Paediatrics, Adolescent Medicine and Neonatology, Faculty of Medicine, Medical Center, University of Freiburg, 79106 Freiburg, Germany; 16 Norwich Research Park, Quadram Institute Bioscience, Norwich, NR4 7UQ UK; 17 Consultant Orthopaedic Surgeon, Department of Trauma & Orthopaedics, Addenbrooke’s, Cambridge University Hospitals NHS Trust, Cambridge, UK

**Keywords:** Vitamin B12, Plant-based diets, Public health, Planetary health, Dietary recommendations, Vegetarian and vegan populations, Women of child-bearing age, Pregnancy

## Abstract

Vitamin B_12_ is an essential nutrient that is not made by plants; consequently, unfortified plant-based foods are not a reliable supply. Recent estimates suggest high rates of vitamin B_12_ deficiency among the vegetarian and vegan populations, particularly in pregnant women or women of child-bearing age who, for ethical and health reasons, are shifting towards higher consumption of plant-based foods in ever-increasing numbers. Vitamin B_12_ plays crucial metabolic roles across the life-course and in particular during pregnancy and in early development (first 1000 days of life). Evidence now implicates vitamin B_12_ deficiency with increased risk to a range of neuro, vascular, immune, and inflammatory disorders. However, the current UK recommended nutrient intake for vitamin B_12_ does not adequately consider the vitamin B_12_ deficit for those choosing a plant-based diet, including vegetarianism and in particular veganism, representing a hidden hunger. We provide a cautionary note on the importance of preventing vitamin B_12_ deficits for those individuals choosing a plant-based diet and the health professionals advising them.

## Key Recommendations to prevent vitamin B12 deficits for individuals choosing a plant-based diet:

**Table:** 

A daily vitamin B12 supplement taken with other foods for optimal absorption.
Check food packaging labels for vitamin B12 fortified products when opting for plant-based animal alternative diets.
Be aware that vitamin B12 deficiency can occur without developing anaemia and often neurological symptoms are more commonly observed (fatigue, memory impairment, cognitive changes, and depression).
Have your blood vitamin B12 levels monitored, particularly if no B12-containing supplements have been taken in the last 3-6 months.
Seek expert advice to support planning a plant-based diet or if you are: [a] transitioning to a vegan diet, [b] planning to become pregnant, or [c] older than 60 years of age.

## Introduction

Plant-based diets, in any form, are becoming increasingly common in Western society [1], whether motivated by environmental and/or animal welfare concerns or simple dietary preferences. Dietary trends, however, should be considered in the broader context of overall nutrition, where both undernutrition and overnutrition increasingly have adverse impacts on both public and planetary health, termed “The Global Synergy” [2]. Given the steep population rise and inevitable strains on global food supply (e.g., it is anticipated that by 2050, the supply of animal-based products will need to rise by 44% to sustain demands based on current global consumption), plant-based diets offer a potentially healthier and more sustainable solution [3].

**Fig. 1:**
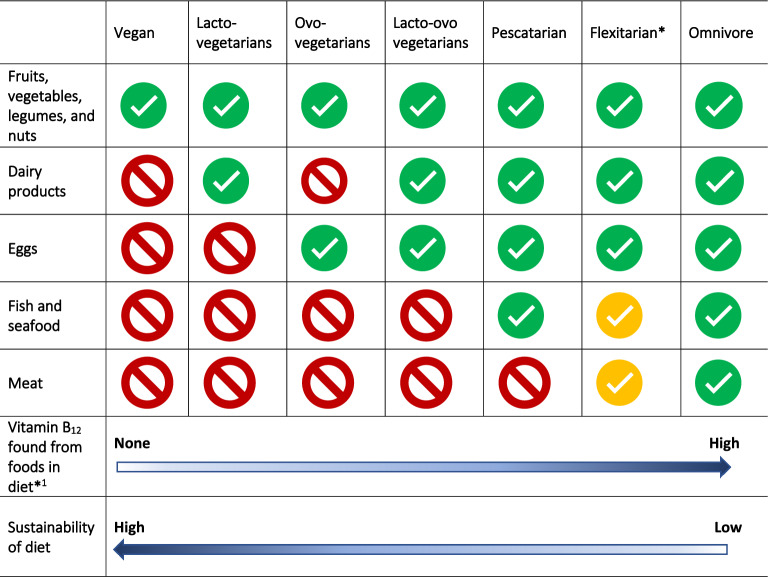
Presents the pattern of consumption for different food components ranging from a vegan to omnivore diet. *A flexitarian diet may occasionally consume fish, seafood and animal products but likely limit their consumption of these foods for environmental and health reasons. *.^1^Shows the gradient of vitamin B_12_ found in foods from differing diets, ranging from none in vegan diets to high in omnivore (without the intake of supplements or fortified foods). Fig. 1 has taken inspiration from Fig. 1 in review [19]

The adoption of sustainable diets that have a positive planetary impact implicate a reduced consumption of animal produce [4] and with it, an insufficient intake of vitamin B_12_ (< 4–20 µg/day). For example, the EAT-Lancet report recommends a 70% reduction in meat consumption compared to the standard omnivore diet in the UK, and a move towards more diverse largely plant-based diets [5]. The consequent drop in vitamin B_12_ intake in increasingly sustainable diets is more rapid and severe if the person moves to a vegan diet (and faster still if they transition from vegetarianism to veganism). Dietary vitamin B_12_ intake decreases with a greater plant-based diet, with average daily B_12_ intake estimated to be 7.2 µg in meat-eaters but only 0.4 µg in vegans [6]. The overall reduced intake of animal produce and consequently vitamin B_12_ suggests a hidden hunger, referring to the phenomenon of inadequacy or imbalance of one or more nutrients in diet, despite eating plenty of food. A spectrum of opportunity exists wherein individuals can contribute to sustainability (consuming less animal products and more plant-based foods, Fig. 1) while minding the importance of monitoring and supplementing with vitamin B_12_ to prevent its deficiency across the life-course.

### Definitions of plant-based diets

Various forms of plant-based diets exist, offering consumers different options depending upon the food groups that are being excluded. One of the strictest forms of plant-based diet is a vegan diet, which eliminates all animal foods (both meats and products, such as milk, eggs, and cheese), along with less obvious by-products, including gelatine and honey. Other plant-based diet categories include lacto-ovo-vegetarians, who eat dairy and eggs but exclude meat and fish, ovo-vegetarians who include eggs but exclude all other animal products, lacto-vegetarians who consume dairy products but exclude all other forms of animal products (including eggs), and pescatarians who eat fish and shellfish [7]. In addition, other popular terms are emerging, with some individuals fluidly labelling themselves as flexitarian, occasionally eating fish or animal products yet primarily following a plant-based diet. Figure 1 presents the described combination of food intake in differing diets. With a greater inclusion of unfortified plant-based foods, diets become more sustainable yet poorer in vitamin B_12_ content.

### Factors that underpin plant-based diets as a food choice

Apart from cultural influences, the main factors that govern intentions to follow a plant-based diet include ecological, ethical, and health concerns [8, 9]. There is also a growing influence of perceived societal norms as a factor underpinning plant-based diets as a food choice, adding to the pressure to preserve planetary and human health [10]. These trends in turn affect the food industry in high-income countries, where the plant-based sector has seen a 49% increase in sales since 2018 [3]. This upsurge in the availability and visibility of plant-based alternatives [e.g., for sources of protein], with shifts in societal norms and lifestyle, have contributed to a cultural shift towards consuming plant-based foods and fewer animal products, perceived as being more viable, healthier, sustainable, and more economical [11].

### Dietary planning and risk of vitamin B_12_ deficiency in plant-based diets

In the 2018 UK Food Standards Agency’s ‘Food and You’ survey, 3% of participants self-identified as vegetarian and 1% as vegan [12]. Importantly, a similar profile is also seen in Western Europe and North America [8, 13, 14].

Below are quotes from different global societies of Nutrition and Dietetics, which have published their expert position statements on plant-based diets.

**UK**—“Carefully planned plant-based diets can support healthy living at every age and life stage. Plant-based diets can help to manage weight and may reduce the risk of type 2 diabetes and other chronic diseases. You can get all essential nutrients from plant foods but vegans need to ensure a reliable source of vitamin B_12_.*”* [7]

**US**—“It is the position of the Academy of Nutrition and Dietetics that appropriately planned vegetarian, including vegan, diets are healthful, nutritionally adequate, and may provide health benefits in the prevention and treatment of certain diseases. These diets are appropriate for all stages of the life cycle, including pregnancy, lactation, infancy, childhood, adolescence, older adulthood, and for athletes. Plant-based diets are more environmentally sustainable than diets rich in animal products because they use fewer natural resources and are associated with much less environmental damage”…… “Vegans need reliable sources of vitamin B_12_, such as fortified foods or supplement”- [14]

**France**—[Bulletin French Academy of Medicine]: “Vegetarian diets increase the risk of vitamin B_12_ deficiency, mainly in infants of vegetarian mothers, pregnant women, and the elderly. The effects of vegetarian diets on B_12_ status in these age groups requires special attention. On the other hand, there is a protective effect of vegetarian diets on the risk of pathological obesity, of the other components of the metabolic syndrome including diabetes and hypertension and on the risk of cardiovascular diseases” [15].

**Italy**—“Well-planned vegetarian diets that include a wide variety of plant foods, and a reliable source of vitamin B_12_, provide adequate nutrient intake. Government agencies and health/nutrition organizations should provide more educational resources to help Italians consume nutritionally adequate vegetarian diets” [16]

**Germany** [childhood and adolescence]—“Vitamin B_12_ should be supplemented in people of all age groups who follow a strict vegan diet without consuming animal products. A vegetarian diet in childhood and adolescence requires good information and supervision by a paediatrician, if necessary, in cooperation with an appropriately trained dietary specialist.”[17]

**Spain** [infants and children]—“A vegetarian or a vegan diet, as in any other kind of diet, needs to be carefully designed. After reviewing current evidence, even though following a vegetarian diet at any age does not necessarily mean it is unsafe, it is advisable for infant and young children to follow an omnivorous diet or, at least, an ovo-lacto-vegetarian diet.”

They also mention the need to use B_12_ supplements at all ages, as well as other nutrients (iodine, iron, vitamin D_3_, and poly-unsaturated fatty acid n-3), when required [18].

All these statements identify vitamin B_12_ deficiency as a risk to health and wellbeing in both vegetarian and vegan diets and clearly state that adopting such diets, especially a vegan diet, requires adequate planning and continuous monitoring. Among the statements, Spain and Germany adopt the most conservative position, in particular warning against adherence to vegan diets in children. The UK statement is less explicit, which is in line with the general UK approach to recommendations for B_12_ (see later). Nonetheless, they all emphasise proper dietary planning and regular monitoring as being a sensible precaution. In agreement with the statements, we highlight the need to seek expert advice on how to carefully plan and implement a healthful vegan diet.

### Vegan diet in health and disease

Regarding vitamin B_12_, we believe a cautionary note and further clarity is required to accompany the health benefits associated with plant-based diets, with particular attention to vegan diets.

Vegetarian and vegan diets are associated with beneficial effects on the blood lipid profile and a reduced risk of negative health outcomes, including diabetes, ischemic heart disease, and cancer risk [19]. While it is commonly recognised that a healthy vegan diet is associated with better general health, independent studies on all-cause mortality have yielded contrasting results [20–23]. Notably, there is increased risk of micronutrient deficiencies within an unsupplemented vegan diet, potentially offsetting any health benefits; however, more comprehensive studies are required to establish this relationship [13, 24–27]. Specifically, an unsupplemented diet lacks adequate amounts of a number of micronutrients and minerals that are insufficiently found in plants or may have low(er) bioavailability. These include iron, calcium, iodine, and selenium [13, 19], but the best example is offered by vitamin B_12_, also known as cobalamin. This complex compound is only found in substantial quantities in animal products, passed through the food chain from the bacteria that make it. In contrast, plants neither need nor synthesise vitamin B_12_. Although the nutrient is often detected routinely in algae, that is seaweeds (or sea vegetables) such as nori or microalgal products, this is again as a result of association with B_12_-producing microbes [28]. As a consequence, the bioavailability of vitamin B_12_ may differ from each batch sold, making them an unreliable source of intake. Consequently, the only reliable food sources containing vitamin B_12_ are from animal produce and adequately fortified plant-based foods.

Vitamin B_12_ is an integral cofactor for two vital cellular metabolic reactions [29] and is essential for the synthesis of blood cells and brain nerve tissue [30]. Low vitamin B_12_ status and overt vitamin B_12_ deficiency among vegans and vegetarians are more commonly observed and reported, largely due to low dietary exposure [13, 19, 25]. Individuals who adhere to a vegan and vegetarian diet since birth show higher rates of vitamin B_12_ deficiency in comparison to vegetarians who adopt such a diet later in life [19, 33]. Moreover, existing data show a greater rate of vitamin B_12_ deficiency among vegans than vegetarians, although prevalence among vegetarians is still substantial [34].

A vegan diet is associated with higher fracture risk, likely due to low BMI (Body Mass Index) and other nutrient deficiencies [35, 36]. Metabolic signs of vitamin B_12_ deficiency may be associated with accelerated bone turnover in those following a vegetarian diet, potentially causing adverse effects on bone health [37]. In addition, there is some evidence showing an adverse relationship between mental health and a vegan and vegetarian diets, particularly depression [38]. Low vitamin B_12_ status is associated with risk of developing neuropsychiatric and neurological disorders [39]

### Vitamin B_12_ deficiency

It is currently unknown how long it takes for vitamin B_12_ deficiency to occur in individuals adopting a vegan diet. At first, the symptoms may be subtle and often ascribed to stress or other lifestyle events [40]. Even as more symptoms manifest, they may be misinterpreted, because vitamin B_12_ deficiency can occur without serum levels being below the usual diagnostic cut-off for ‘deficiency’ or without an associated diagnosis of megaloblastic anaemia [31]. Signs and symptoms include cognitive changes (such as depression, memory impairment, confusion, psychosis, and tiredness) and dyspnoea, whilst the neurological complications may cause loss of sensation, postural hypotension, muscle weakness, or loss of mental and physical drive [41, 42]. In any diet, low vitamin B_12_ status warrants attention given its association with increased risk of a myriad of clinical consequences, including the neurological conditions noted above. It is also associated with pregnancy complications, including developmental anomalies, spontaneous abortions, preeclampsia, and low birth weight (< 2500 g)[43, 44]. Adequate vitamin B_12_ status periconceptionally and during pregnancy is vital for neural myelination, brain, and cognitive development and growth in the infant. Deficiency during these critical times may result in adverse effects that may be irreversible [45]. In older adults, vitamin B_12_ deficiency is more commonly observed due to the high rate of atrophic gastritis, accompanying vitamin B_12_ malabsorption and the increasing incidence of pernicious anaemia with increasing age, which can occur irrespective of their dietary choices [46].

### Current recommendations for vitamin B_12_ in the UK and beyond

The UK's recommended nutrient intake (RNI) for vitamin B_12_ is currently set at 1.5 µg/day for adults and is unaltered for pregnancy [47]. However, this differs in the USA, where the RNI for adults is 2.4 µg/day and is modified for pregnant and lactating women to 2.6 and 2.8 µg/day, respectively [48]. Within the European Union, the estimated average requirement (EAR) is 4 µg/day for adults and increases for pregnant and lactating women to 4.5 and 5 µg/day, respectively [49]. Thus, the current UK recommendations for vitamin B_12_ are both inadequate and incomplete. Not only are they significantly lower than in other developed countries, but they are also unaltered for different at-risk population groups (e.g., women of child-bearing age and pregnant women), which are likely to have substantially higher requirements as defined in the report issued by The Institute of Medicine (US) Standing Committee on the Scientific Evaluation of Dietary Reference Intakes and its Panel on Folate, Other B Vitamins, and Choline [50]. Evidence from clinical studies shows that healthy individuals require a daily intake of approximately 6 µg/day to optimize all biomarkers of vitamin B_12_ deficiency [51, 52]. As outlined in the ESPEN micronutrient guidelines, increased physiological needs for B_12_ occur also with ageing, certain chronic illnesses, and the use of certain medications [53]. Thus, a recommendation of 4–20 µg/day is more appropriate to prevent B_12_ deficiency across the life-course [51, 54–56].

### Biomarkers of vitamin B_12_ status

There are several factors affecting vitamin B_12_ status across the life-course. Serum vitamin B_12_ is the most common way to measure vitamin B_12_ levels [30]. Other serum biomarkers used alone or in combination—low holotranscobalamin (holo-TC) and elevated methylmalonic acid (MMA) and homocysteine concentrations—provide further ways to assess status and detect vitamin B_12_ deficiency [57]. It should be noted, however, that metabolic evidence of vitamin B_12_ deficiency may be found at serum vitamin B_12_ levels up to as high as 350 pmol/L [31]. Previous research has shown that individuals following a vegan diet without consuming vitamin B_12_ supplements (Fig. 1) are at a much higher risk of having low concentrations of serum vitamin B_12_, although this may not equate to cellular deficiency, and measurement of a biomarker of cellular metabolism (such as MMA) is needed to establish actual deficiency [58]. Therefore, it has been suggested to use holo-TC accompanied with MMA or homocysteine as appropriate biomarkers to identify those exhibiting vitamin B_12_ deficiency [59, 60].

### Population groups adhering to a vegan diet at higher risk of vitamin B_12_ deficiency

Women of child-bearing age, pregnant, and lactating women adhering to an unsupplemented vegan diet are at a much higher risk of vitamin B_12_ deficiency, and their offspring are at elevated risk of low birth weight and preterm births [44, 61, 62]. Prevalence of deficiency among vegetarian and vegan pregnant women are estimated to be 17–39% in lower socioeconomic countries [32], but we need current and more representative data, particularly among UK dwelling adults. The most at-risk population are vegans who do not take any form of vitamin B_12_ supplements [33]. There could also be a much greater risk to vegetarian individuals, with already low vitamin B_12_ status, who change to a vegan diet.

### Ensuring adequate B_12_ intake among those adhering to vegan diets

The British Dietetic Association (BDA) advises that those following a vegan diet should use good manufacturing practice (GMP) certified vitamin B_12_ supplements and consume B_12_ fortified foods [7]. GMP certified status supplements prove that supplements meet the highest standards for manufacturing products regulated by the Food and Drug Administration (FDA) and the European Medicines Agency (EMA). Data from the Quadram Institute Food Databanks National Capability from studies of the macro/micronutrient composition of available vegan products in the UK supermarkets have shown that most do not commonly or adequately fortify their products with vitamin B_12_ [Unpublished data: Zhang L, Langlois E, Nikolaeva A, et al. (2021) The macro and micronutrient composition of vegan diet and links to health benefits]. For example, milk substitutes were found to be far less likely to be fortified with vitamin B_12_ than calcium. UK vegans consuming typical [31] vegan diets were found to have dietary vitamin B_12_ intakes considerably lower than recommended levels, consuming only 0.5 ± 0.08 µg/day, approximately 10% of the B_12_ intakes provided in the average UK diet [63]. Overall, a general lack of vitamin B_12_, along with selenium and iodine, was found within plant-based substituted foods currently available on the UK market, indicating that they are generally not reliable alternatives for meeting human vitamin B_12_ requirements. This highlights an urgent demand for mandatory and adequate fortification of plant-based dairy and meat alternative foods in the UK, in particular to combat deficits in vitamin B_12_. Other reports from the Food Databanks National Capability (2019) have shown that currently available animal produce, specifically pork-derived products, have significantly lower vitamin B_12_ and iodine content (around one-third less) compared with the early 1990s [64]. This is most likely due to pigs no longer being fed animal offal, i.e., B_12_ status in animals is lower when provided with less animal-based food. Using supplements within a well-planned vegan diet or plant-based diet is likely to provide a more effective and sustainable method to prevent overt vitamin B_12_ deficiency [65]. A few studies have investigated the rate of vitamin B_12_ supplementation practices among sub-groups following vegan diets; this is an area for future investigation as these sub-groups are most at risk.

## Conclusions

Vitamin B_12_ is an essential nutrient that is absent from unfortified plant-based foods. We have aimed to provide a cautionary note on the importance of vitamin B_12_ to those individuals seeking to adopt a plant-based diet and in particular a vegan diet. An appropriately planned vegan diet has the potential to uphold a healthy and sustainable life, but consideration of a complete diet is essential to ensure the adequate provision of this limiting nutrient. Adverse health effects are associated with long-term inadequate vitamin B_12_ status, which is more commonly observed among those adhering to unsupplemented and unfortified wholly plant-based diets. We specifically highlight women of child-bearing age, pregnant and lactating women, and older adults, as well as those individuals who are already following a plant-based diet (vegetarians) and who transition to a vegan diet, as these population sub-groups are most at risk. Prevention of vitamin B_12_ deficiency through supplementation offers an effective, economical, and sustainable way to avoid the adverse health consequences in such situations. We, therefore, encourage people planning for a vegan diet to take a certified supplement of vitamin B_12_ at a mealtime and seek professional guidance should any symptoms relating to a possible vitamin B_12_ deficiency occur. Considering the above caution, we support well-planned plant-based diets enhanced with vitamin B_12,_ which have the ability to positively impact both human and planetary health.
